# Symptomatic intracranial abscess after treating lower cervical spine fracture with halo vest: a case report and review of literature

**DOI:** 10.1186/1757-1626-2-101

**Published:** 2009-01-29

**Authors:** Dimitrios S Evangelopoulos, Panagiotis Kontovazenitis, Konstantinos Kokkinis, Nikolaos Efstathopoulos, Dimitrios Korres

**Affiliations:** 1 Orthopaedic Dept, University of Athens, KAT Accident's Hospital, Athens, Greece; 2 Radiology Dept, KAT Accident's Hospital, Athens, Greece; 3 Orthopaedic Dept, University of Athens, Konstantopoulion Hospital, Athens, Greece; 4 Orthopaedic Department, University of Berne-Inselspital, Berne, Switzerland

## Abstract

We present the case of a nineteen year old male, who sustained a fracture of anterior-superior surface of C7, combined with anterior subluxation at the level of C6–C7 vertebrae. After x-ray and CT examination, he was treated conservatively by a Halo-vest. After mobilization, the patient was discharged from the hospital with instructions to visit the outpatient's clinic at regular bases.

Despite of our instructions, he did not attend the regular follow-up and, three months later, he visited the emergencies complaining of pin loosening and serious headaches. He was admitted to the clinic in order to perform blood tests and new radiological control. During the first day, high fever (over 38,5°C) was added to his symptoms. Blood exams were indicative of inflammation. Further investigation with CT-scan revealed the presence of a subdural abscess. After consulting the neurosurgeon, the patient was treated conservatively with antimicrobial drugs. Three weeks later he returned home without any symptoms. Since then, he is visiting regularly our clinic and no problems occurred during follow-up.

## Introduction

Injuries of the subaxial cervical spine (C3–7) are among the most common and potentially most devastating injuries involving the axial skeleton. The age distribution for such lesions follows a characteristic pattern: in younger people, it is usually the result of high-energy trauma while in older people it is mainly caused by the exertion of a low-energy force[1,2]. Neurological deficit is present in approximately 40% of patients[2] and in approximately 10% of traumatic cord injuries, no radiographic evidence is revealed. Treatment choices for such lesions include prolonged bed rest, orthotic support and internal surgical stabilization [3-5]. In this case, our patient was treated conservatively by means of a Halo vest[6,7]. Despite its minimal invasiveness, application of such a device may lead to very rare but serious complications.

## Case presentation

We present the case of a nineteen year old male with a motorcycle accident, admitted to the emergencies complaining about a mild pain at the base of the neck. No other serious injuries were present despite of the severity of the crash.

A free medical history was reported. Clinical examination confirmed patient's stable respiratory and hemodynamic condition, the lower cervical pain as well as the absence of any neurological deficit. X-ray examination demonstrated a small fracture at the anterior superior surface of the C7 vertebrae indicative of a combination of flexion-destruction forces applied during the accident.

Dynamic cervical spine x-rays were indicative of instability and CT-scan 3D analysis, performed to evaluate x-ray findings, confirmed the presence of the fracture and the anterior subluxation at the C6–C7 level.

**Figure 1:**
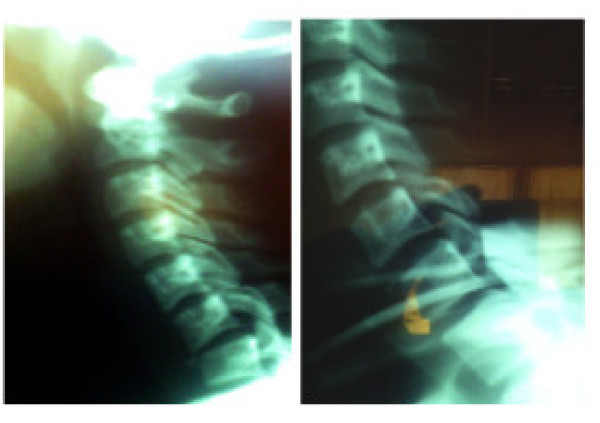
**Lateral pre and post reduction x-rays**.

Conservative treatment with Halo-vest, x-ray evaluation of the reduction and a new neurological examination were performed, figure 1. Patient was discharged from the hospital with instructions to visit the outpatients department according to treatment schedule.

Despite of our instructions, the patient neglected visiting regularly the outpatient department and presented three months later to the emergencies complaining of mild headaches and fatigue.

Clinical examination revealed mild redness and edema of the skin as well as pin loosening and irritation at the point of pin insertion. GCS was estimated at 15/15 and no neurological deficit was detected. Lateral and anteroposterior X-rays were normal. Blood count presented an increased amount of WBCs, mainly neutrophils. Pins were tightened and patient was instructed to visit the clinic the following day for new blood and radiologic exams.

The following day, the patient was admitted in our clinic. New blood counts and x-rays were performed and samples were taken from the area of pin insertion with minimal incision and were sent to the Microbiology Department. Due to the lack of definite diagnosis, further radiological and serological tests were performed. CT-scan revealed the presence of a small subdural abscess. Immediate halo removal, Philadelphia cast application and i.v. antibiotic administration (Rocephin: IIIrd generation cephalosporine) were performed.

**Figure 2:**
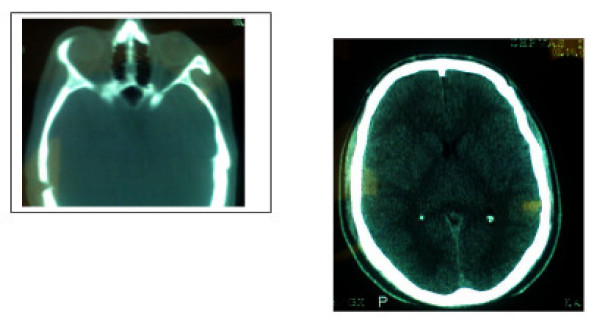
**Pre-treatment/post-treatment CT scan**.

During the first day, our patient presented high fever (over 38,5°C), vomiting, decrease in GCS, gradually developing signs and symptoms of meningitis. Symptoms of an epileptic seizure were manifested during the first night at the hospital. After consulting the neurosurgeons, new blood counts, BSR, CRP, blood cultures and ONP were performed and CSF was sent for culture. Antiepileptic drugs (Depakine) were also added to patient's treatment. Since a skin inflammation was diagnosed at the point of pin insertion, the other systems showed no signs of inflammation (respiratory tract, urinary tract, GI system, cardiovascular system etc) and the neurological profile did not mach with our CT-scan findings, we proceeded to a cerebral MRI in order to investigate the extent of the inflammation. MRI indicated two subdural abscesses at the parietal lobe, near the transverse sinus, pressing the walls of the meninges. Cultures from CSF and from the point of pin insertion isolated Staphylococcus MRSA and proper antimicrobial drugs were administered to the patient (Vancomycin, Ciprofloxacin). Conservative treatment was suggested by the consulting neurosurgeon and therefore intravenous antibiotics were administered for six weeks under continuous neurological and serological follow-up. Moreover, cerebral CT-scans were performed in order to evaluate treatment's efficiency. All neurologic symptoms resolved after treatment and no other tonic-clonic seizure occurred, figure 2.

## Discussion

Comparing to the upper part, lower cervical spine possesses a homologous anatomic pattern. This fact has a direct effect on the type of lesions observed in this particular area, leading mainly to two important problems: the ligamentous-osseous instability and the neurological deficit.

Conservative treatment, either by means of traction followed by a Philadelphia cast or by means of a Halo Vest, for stable and certain unstable lesions, is well documented in the literature. Despite of its minimal invasiveness, the application of a Halo vest is associated with minor or major complications such as pin site infections leading to superficial or deep infections. Various other minor complications that have been reported in the literature include pressure sores, nerve injury, dysphagia, severe scars. Reported major complications, include cranial osteomyelitis, abscess formation, halo dislodgement and loss of fracture alignment or reduction[8].

Several authors[9-12] have reported asymptomatic brain abscesses as a result of a halo orthosis.

Goodman et al. [13] reported four cases of asymptomatic brain abscess. At least two of them were associated with tightening of the halo loosened screws.

Rosenblum et al[14] published the case of a 23 year old male with spinal cord injury that developed extreme agitation and psychosis after halo application, due to an intracranial abscess. Patient's pathologic behavior resolved after receiving treatment for the abscess. This was the first report in the literature for a symptomatic abscess after halo vest treatment. In 2001, Papagelopoulos et al[15] reported intracranial pin penetration and asymptomatic epidural abscess formation in a patient with medical history of cranioplasty and ankylosing spondylitis. Kingma et al[16], in 2006, reported the case of a 76 year-old male with ankylosing spondylitis and an unstable fracture at the level of the 6^th ^and 7^th ^cervical vertebrae, treated conservatively with a halo vest. Eight weeks post application pin loosening and headaches appeared combined with an epileptic seizure. MRI confirmed the diagnosis and after conservative treatment, no other seizures occurred. Saeed et al [17] in 2007, reported a case of a brain abscess after halo treatment, causing right-sided headaches fourteen days post application. After halo removal he noted purulent discharge from the right occipital pin side, worsening headaches and associated nausea and vomiting. Symptoms resolved after surgical abscess evacuation and proper intravenous treatment.

## Conclusion

Our medical staff is familiar with the use of Halo since this device has been often used in the conservative treatment of cervical spine fractures. During these years no serious complications occurred. All our patients are instructed to visit the outpatient clinic at regular bases for clinical and x-ray examination. Rare minor complications, mainly superficial pin site infections, are treated conservatively by means of oral antibiotics. Early removal of the device due to infection has never been performed in any of our patients.

An orthopaedic surgeon should always bear in mind that conservative treatment with minimal invasive devices even in experienced hands may lead to life threatening complications. The possibility of such complications should always be taken into consideration when dealing with such devices and extreme consciousness and proper follow-up are mandatory in order to be able to deal successfully such serious problems.

## Consent

Written informed consent was obtained from the patient for publication of this case report and any accompanying images. A copy of the written consent is available for review by the Editor-in-Chief of this journal.

## Competing interests

The authors declare that they have no competing interests.

## Authors' contributions

EDS diagnosed, treated the patient post admittance in the clinic and wrote the manuscript. KP treated the patient post admittance and collected data. KK performed the CT-scans and MRI. EN supervised treatment and reviewed manuscript. KD advised and supervised treatment and reviewed manuscript. All authors read and approved the final manuscript.
